# Phosphate is a potential biomarker of disease severity and predicts adverse outcomes in acute kidney injury patients undergoing continuous renal replacement therapy

**DOI:** 10.1371/journal.pone.0191290

**Published:** 2018-02-07

**Authors:** Su-Young Jung, Jaeyeol Kwon, Seohyun Park, Jong Hyun Jhee, Hae-Ryong Yun, HyoungNae Kim, Youn Kyung Kee, Chang-Yun Yoon, Tae-Ik Chang, Ea Wha Kang, Jung Tak Park, Tae-Hyun Yoo, Shin-Wook Kang, Seung Hyeok Han

**Affiliations:** 1 Department of Internal Medicine, College of Medicine, Institute of Kidney Disease Research, Yonsei University, Seoul, Korea; 2 Department of Internal Medicine, NHIS Medical Center, Ilsan Hospital, Ilsan, Korea; National Yang-Ming University, TAIWAN

## Abstract

Hyperphosphatemia is associated with mortality in patients with chronic kidney disease, and is common in critically ill patients with acute kidney injury (AKI); however, its clinical implication in these patients is unknown. We conducted an observational study in 1144 patients (mean age, 63.2 years; male, 705 [61.6%]) with AKI who received continuous renal replacement therapy (CRRT) between January 2009 and September 2016. Phosphate levels were measured before (0 h) and 24 h after CRRT initiation. We assessed disease severity using various clinical parameters. Phosphate at 0 h positively correlated with the Acute Physiology and Chronic Health Evaluation II (APACHE II; *P* < 0.001) and Sequential Organ Failure Assessment (SOFA; *P* < 0.001) scores, and inversely with mean arterial pressure (MAP; *P* = 0.02) and urine output (UO; *P* = 0.01). In a fully adjusted linear regression analysis for age, sex, Charlson comorbidity index (CCI), MAP, and estimated glomerular filtration rate (eGFR), higher 0 h phosphate level was significantly associated with high APACHE II (*P* < 0.001) and SOFA (*P* = 0.04) scores, suggesting that phosphate represents disease severity. A multivariable Cox model also showed that hyperphosphatemia was significantly associated with increased 28-day (HR 1.05, 95% CI 1.02–1.08, *P* = 0.001) and 90-day (HR 1.05, 95% CI 1.02–1.08, *P* = 0.001) mortality. Furthermore, patients with increased phosphate level during 24 h were at higher risk of death than those with stable or decreased phosphate levels. Finally, *c*-statistics significantly increased when phosphate was added to a model that included age, sex, CCI, body mass index, eGFR, MAP, hemoglobin, serum albumin, C-reactive protein, and APACHE II score. This study shows that phosphate is a potential biomarker that can reflect disease severity and predict mortality in critically ill patients receiving CRRT.

## Introduction

Acute kidney injury (AKI) is common in critically ill patients. Approximately 5% of patients with AKI in the intensive care unit (ICU) require renal replacement therapy (RRT), [1] and these patients are more likely to have higher mortality and to progress to chronic kidney disease (CKD) [2] than those without AKI [3–5]. Thus, identification of risk factors is important for predicting adverse outcomes. However, there is no single biomarker to reliably establish cost-efficient risk stratification in this devastating condition.

Electrolyte and mineral imbalances are common even in patients receiving RRT. Disturbances in the regulatory mechanisms can result in significant consequences, especially in critically ill patients [6]. Phosphate is the most abundant intracellular anion in the body and is an important component in multiple physiologic processes affecting many different organ systems [7]. Elevated serum phosphate levels are usually found in patients with moderate to severe CKD, and are associated with cardiac valvular and vascular calcification, thus leading to increased cardiovascular events and mortality [8, 9]. Hyperphosphatemia is also a common condition in patients with AKI. It is likely due to decreased phosphate removal and secondary hyperparathyroidism as a result of reduced kidney function [10, 11].

To date, the importance of hyperphosphatemia has been largely emphasized in patients with CKD. However, studies on the clinical implication of hyperphosphatemia in critically ill patients with AKI are lacking. We previously showed that increased phosphate levels predict poor prognosis in patients with septic AKI receiving CRRT better than do other electrolytes and minerals [10]. This finding led us to hypothesize that phosphate may correlate with the degree of illness and serve as a marker of disease severity. Therefore, we conducted an observational study to evaluate the association between hyperphosphatemia and clinical parameters reflecting disease severity, and to test whether phosphate levels are helpful in risk stratification in these patients.

## Materials and methods

### Study population

Data were retrieved from the medical records of 1144 patients who received CRRT in the ICU at Yonsei University Health System Severance Hospital and National Health Insurance Service Medical Center Ilsan hospital between January 2009 and September 2016. Among 2391 patients who were initially assessed for study eligibility. Patients with stage 2 according to the Acute Kidney Injury Network (AKIN) criteria [12] or more (>2-fold increase in the serum creatinine or urine output [UO] <0.5 mL∙kg^-1^∙h^-1^ for 12 h) were eligible for the study. We excluded patients who met the following criteria: age <18 years, pregnancy or lactation, history of CKD or dialysis or CRRT before the study, postrenal obstruction and prior kidney transplantation. Thus, a total of 1144 patients were included in the analysis.

**Fig 1:**
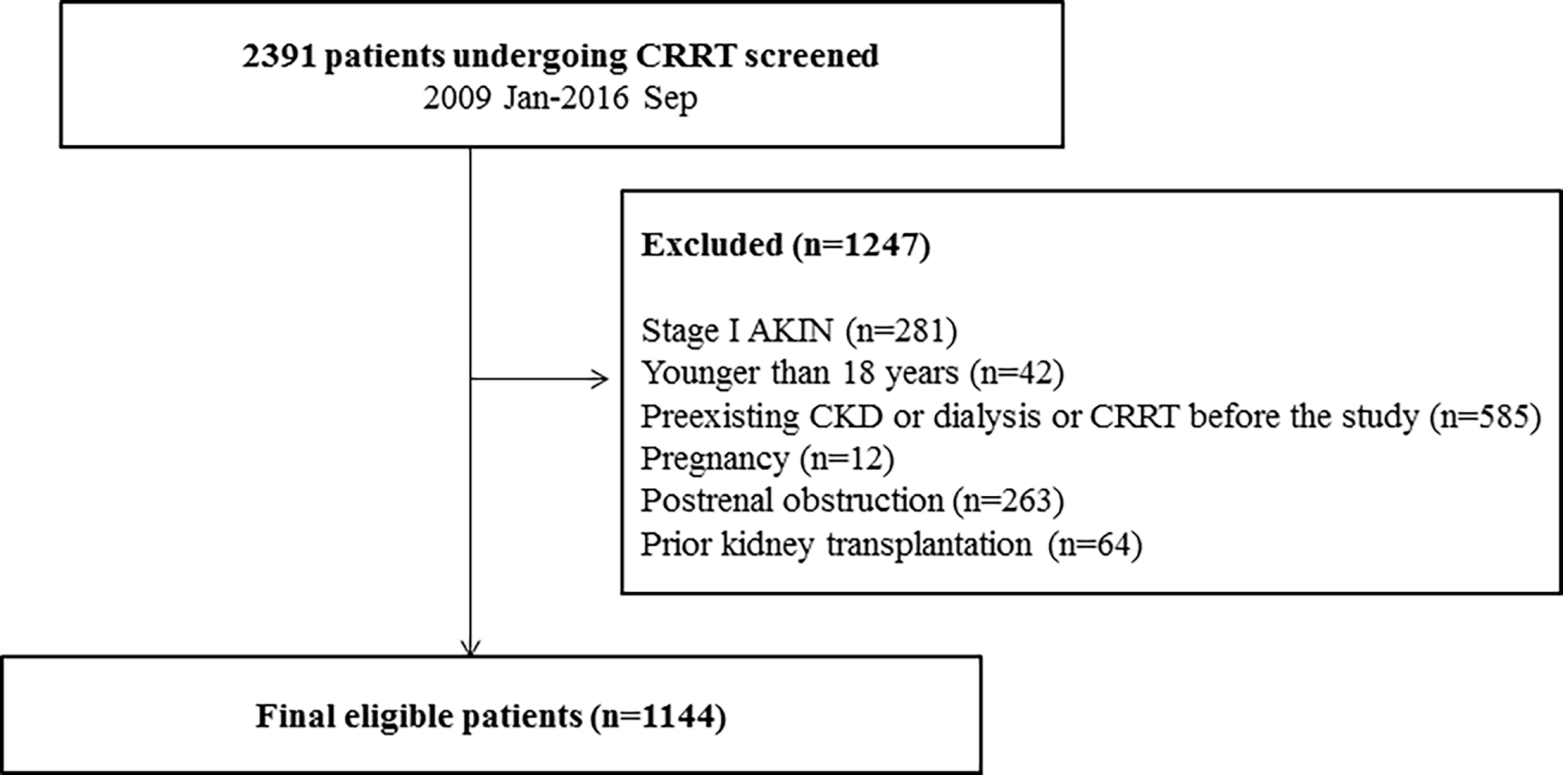
Flow chart of patient selection. CRRT continuous renal replacement therapy, AKIN acute kidney injury network, CKD chronic kidney disease.

The study was approved by Yonsei University Health System, Severance Hospital, Institutional Review Board and followed the provisions of the Declaration of Helsinki (approval no. 4-2016-1073). Because the current study was a retrospective, medical-record-based study and the study subjects were de-identified, the board waived the need for written consent from the patients. (Fig 1)

### Clinical and biochemical data collection

Before starting CRRT, demographic and clinical data including age, sex, body mass index, and comorbidities were recorded. Phosphate levels were measured before starting CRRT (0 h) and 24 h after CRRT initiation. We also collected the following biochemical laboratory data at 0 h: hemoglobin, white blood cell, serum creatinine, albumin, bicarbonate, potassium, lactate, aspartate aminotransferase, alanine transaminase, and total bilirubin levels. The reference range of phosphate level was 2.5–4.5 mg/dL, and hyperphosphatemia was defined as phosphate level >4.5 mg/dL. We also collected data for age-adjusted Charlson comorbidity index (CCI), Sequential Organ Failure Assessment (SOFA) score, and Acute Physiology and Chronic Health Evaluation II (APACHE II) score.

Disease severity was assessed by using various clinical parameters: SOFA score, APACHE II score, mean arterial pressure (MAP), UO, norepinephrine dose, C-reactive protein (CRP), and lactate.

### CRRT protocol

Upon the development of AKI in ICU patients, nephrologists decided whether or not to initiate CRRT in those who were critically ill. General indications were sustained oliguria, uncontrolled volume overload, intractable hyperkalemia or metabolic acidosis. Patients received continuous veno-venous haemodiafiltration through the internal jugular, subclavian, or femoral vein using the multiFiltrate (Fresenius Medical Care, Bad Homburg, Germany) or the Prismaflex (Baxter International Inc. Lundia AB, Sweden) machine. The applied dialyzers had a surface area of 1.0 to 1.4 m^2^ with a sieving coefficient for albumin and ß2-microglobulin of 0.001 and 0.58 to 0.65, respectively. CRRT was started at a blood flow rate of 100 mL/min, and this was increased up to 150 mL/min. The total effluent volume as a sum of dialysis and replacement dose was targeted to deliver ≥35 mL∙kg^-1^∙h^-1^ in all patients. The mean duration of 1st CRRT was 40 hours.

### Study endpoints

The study endpoint was death that occurred within 28 and 90 days of CRRT initiation.

### Statistical analysis

SPSS for Windows version 23.0 (IBM Corp., Armonk, NY, USA) was used for all statistical analyses. Continuous variables with normal distribution were expressed as the mean ± standard deviation and compared using the Student’s t-test or one-way analysis of variance. Data with skewed distribution were presented as a median with interquartile range. Categorical variables were expressed as a number (percentage) and compared using the chi-squared test. Variables that did not show normal distribution were compared using Mann-Whitney test or Kruskal-Wallis test. The Kolmogorov-Smirnov test was used to examine the normality of the distribution of parameters. Pearson’s correlation coefficient for normally distributed data and Spearman’s rank correlation coefficient for skewed data were determined to examine association between clinical parameters and disease severity score systems such as APACHE II and SOFA scores. We further conducted multiple linear regression analysis to delineate the relationship between phosphate and disease severity. We used R software (version 2.12.1; R Foundation for Statistical Computing) for cubic spline curves analysis. The smoothHR package was used to provide hazard ratio curves that allows for nonlinear relationship between predictor and survival. Cumulative patient survival curves were derived using the Kaplan-Meier method, and differences between curves were analyzed by log-rank test. Cox proportional hazards models were constructed to identify independent association between phosphate level and mortality. Variables with P ≤ 0.10 in univariate analyses and clinically important variables were entered into the models. Results were presented as hazard ratio (HR) and 95% confidence interval (CI). Violation of the proportional hazards assumption was tested by means of inspection of log (-log [survival]) curves. We used R software (version 2.12.1; R Foundation for Statistical Computing) for cubic spline curves analysis. The *smoothHR* package was used to provide hazard ratio curves that allows for nonlinear relationship between predictor and survival. The smoothHR contains functions that provide point wise estimates of Cox model HR curve for continuous predictors as well as the corresponding confidence limits. We also categorized patients into three groups according to the changes in phosphate levels between 0h and 24h and compared death rates and mortality risk using multivariable Cox models. In addition, Harrell’s C index was calculated to compare the discriminatory ability. Variables were sequentially entered in 3 models. Model 1 included age, sex, CCI, BMI, urine output (2 h), MAP, hemoglobin, serum albumin, and CRP. In model 2, SOFA score or APACHE II score was added to Model 2. Finally, serum phosphate was additionally included in Model 3. Furthermore, receiver operating characteristic (ROC) curve analysis was performed to evaluate the prognostic value of phosphate level for mortality using R package survival ROC, version 2.12.1 (R Foundation for Statistical Computing). We measured the AUC; AUCs were calculated and compared using DeLong's method performed within the R package, *pROC*. All probabilities were 2 tailed, and the level of statistical significance was defined as P < 0.05.

## Results

### Baseline characteristics

Table 1 presents the baseline clinical characteristics and laboratory findings of all patients. The most common cause for CRRT was sustained oliguria. The mean age of the participants was 63.2 ± 14.4 years, and 705 (61.6%) were male. Among the 1144 patients, 398 (34.8%) had diabetes and 186 (16.3%) had heart failure. The mean creatinine levels at admission to hospital and at the time of CRRT initiation were 1.0 (0.7–1.3) and 2.7 ± 1.6 mg/dL, respectively. The mean CCI was 3.2 ± 2.3. The mean SOFA score and APACHE II score was 12.1 ± 3.6 and 27.3 ± 8.0, respectively. The MAP at 0 h was 77.4 ± 14.6 mmHg. A total of 898 (78.5%) patients received mechanical ventilation. The mean white blood cell counts and CRP levels were 11,660 (6452–18,645)/μL and 78.7 (21.0–170.9) mg/L, respectively (Table 1). The mean phosphate level at 0h was 5.7 ± 2.4 mg/dL. Hyperphosphatemia was found in 721 (67.5%) patients before starting CRRT, and remained in 399 (40.6%) at 24 h after CRRT initiation. In contrast, 35 (3.3%) and 115 (11.7%) patients had hypophosphatemia at 0 and 24 h, respectively.

**Table 1:** Baseline characteristics.

Patient Characteristics	Total (n = 1144)	Survivors (n = 323)	Non-survivors (n = 821)		*P*
Age (years)	63.2 ± 14.4	62.6 ± 15.1	63.4 ± 14.1		0.62
Male (%)	705 (61.6)	196 (60.7)	509 (62.0)		0.36
Hypertension (%)	601 (52.5)	204 (63.2)	397 (48.4)		<0.001
Diabetes mellitus (%)	398 (34.8)	131 (32.9)	267 (32.6)		0.01
Heart failure (%)	186 (16.3)	58 (18.0)	128 (15.6)		0.19
Myocardial infarction (%)	112 (9.8)	36 (11.1)	76 (9.3)		0.20
Cerebrovascular disease (%)	114 (10.0)	37 (11.5)	77 (9.4)		0.17
COPD (%)	80 (7.0)	29 (9.0)	51 (6.2)		0.07
MV (%)	898 (78.5)	217 (67.2)	681 (83.0)		<0.001
CCI	3.2 ± 2.3	2.7 ± 2.1	3.4 ± 2.3		<0.001
Cause of AKI					0.15
Sepsis	798 (69.8)	213 (65.9)	585 (71.3)		0.05
Nephrotoxin	37 (3.2)	13 (4.0)	24 (2.9)		0.22
Ischemia	98 (8.6)	26 (8.0)	72 (8.8)		0.40
Surgery	94 (8.2)	36 (11.1)	58 (7.1)		0.02
Others	117 (10.2)	35 (10.8)	82 (10.0)		0.37
Cause of CRRT					0.04
Volume overload (%)	160 (13.9)	55 (17.0)	105 (12.8)		0.04
Metabolic acidosis (%)	242 (21.2)	55 (17.0)	187 (22.8)		0.02
Hyperkalemia (%)	58 (5.1)	12 (3.7)	46 (5.6)		0.12
Uremia (%)	115 (10.1)	37 (11.5)	78 (9.5)		0.19
Oliguria (%)	294 (25.7)	93 (28.8)	201 (24.5)		0.08
Others (%)	275 (24.0)	71 (22.0)	204 (24.8)		0.17
Duration from diagnosis of AKI to CRRT (h)	1.1 [0.3–5.4]	0.9 [0.1–2.6]	1.3 [0.3–6.7]		0.01
AKIN stages					0.46
Stage 2 (%)	298 (26.0)	83 (25.7)	215 (26.2)		
Stage 3 (%)	846 (74.0)	240 (74.3)	606 (73.8)		
BMI (kg/m^2^) at ICU admission	23.8 ± 4.6	24.4 ± 4.3	23.6 ± 4.7		0.01
SOFA score	12.1 ± 3.6	10.2 ± 3.4	12.9 ± 3.3		<0.001
APACHE II score	27.3 ± 8.0	25.0 ± 7.9	28.2 ± 7.8		<0.001
SBP (mmHg)	112.0 ± 21.2	118.3 ± 21.2	109.5 ± 20.6		<0.001
DBP (mmHg)	60.3 ± 14.2	62.8 ± 14.5	59.4 ± 14.0		<0.001
MAP (mmHg)	77.4 ± 14.6	81.3 ± 15.2	75.9 ± 14.1		<0.001
Hemoglobin (g/dL)	9.6 ± 2.2	10.0 ± 2.3	9.5 ± 2.2		<0.001
White blood cell (μL)	11660 [6452–18645]	13385 [9035–20327]	11095 [5247–17910]		<0.001
Albumin (g/dL)	2.6 ± 0.6	2.8 ± 0.6	2.5 ± 0.5		<0.001
Potassium (mEq/L)	4.7 ± 1.1	4.7 ± 1.0	4.7 ± 1.1		0.69
Bicarbonate (mEq/L)	16.9 ± 5.7	17.0 ± 5.1	16.9 ± 6.0		0.71
BUN (mg/dL)	55.8 ± 30.0	51.5 ± 27.3	57.5 ± 30.8		0.003
Phosphate (mg/dL)	5.7 ± 2.4	5.4 ± 2.4	5.9 ± 2.4		<0.001
Creatinine (mg/dL)	2.7 ± 1.6	3.0 ± 1.9	2.6 ± 1.5		<0.001
CRRT dose (ml/kg)	36.5 ± 4.8	36.3 ± 5.0	36.8 ± 4.7		0.001
CRP (mg/L)	78.7 [21.0–70.9]	74.5 [19.2–164.2]	80.4 [21.8–174.7]		0.35

Data are expressed as mean ± standard deviations, median (interquartile range), or number (%).

All laboratory measurements were done at 0 h (before starting CRRT)

*Abbreviations*: *COPD* Chronic obstructive pulmonary disease, *CCI* Charlson comorbidity index, *BMI* Body mass index, *AKIN* Acute kidney injury criteria, *ICU* Intensive care unit, *CRRT* Continuous renal replacement therapy, *eGFR* estimated glomerular filtration rate, *SOFA* Sequential Organ Failure Assessment Score, *APACHE II* Acute Physiology and Chronic Health Evaluation II, *SBP* Systolic blood pressure, *DBP* Diastolic blood pressure, *MAP* Mean arterial pressure, *CRP* C-reactive protein

### Correlation between phosphate levels and disease severity

The SOFA and APACHE II score systems are well-validated tools that can assess disease severity and predict clinical outcome in critically ill patients. Thus, we tested whether phosphate can reflect disease severity. Phosphate level significantly correlated with the APACHE II (*P* < 0.001) and SOFA (*P* < 0.001) scores. We then further investigated the independent association of phosphate level with the SOFA and APACHE II scores. In a multivariable linear regression analysis after adjustment for age, sex, CCI, MAP, and urine volume, phosphate levels were significantly associated with the SOFA (β = 0.10, *P* = 0.02) and APACHE II (β = 0.58, *P* < 0.001) scores (Table 2).

**Table 2:** Univariate and multivariate associations between phosphate level at 0h and disease severity.

	SOFA score				APACHE II score			
	Univariate		Multivariate		Univariate		Multivariate	
Variables	β	*P*	β	*P*	β	*P*	β	*P*
Age	-0.03	<0.001	-0.03	<0.001	0.07	<0.001	0.07	<0.001
Sex	-0.65	0.002	-0.55	0.01	-1.58	0.001	-1.20	0.02
CCI	0.13	0.004	0.09	0.05	0.04	0.69	-0.04	0.70
MAP (mmHg)	-0.02	0.01	-0.02	0.04	-0.06	<0.001	-0.05	0.01
Urine output (2 h)	-0.01	0.001	-0.01	<0.001	-0.01	0.01	-0.004	0.13
Phosphate (mg/L)	0.16	<0.001	0.10	0.02	0.59	<0.001	0.58	<0.001

*Abbreviations*: *CCI* Charlson comorbidity index, *MAP* Mean arterial pressure, *SOFA* Sequential Organ Failure Assessment Score, *APACHE II* Acute Physiology and Chronic Health Evaluation II

### Hyperphosphatemia predicts adverse outcomes

**Fig 2:**
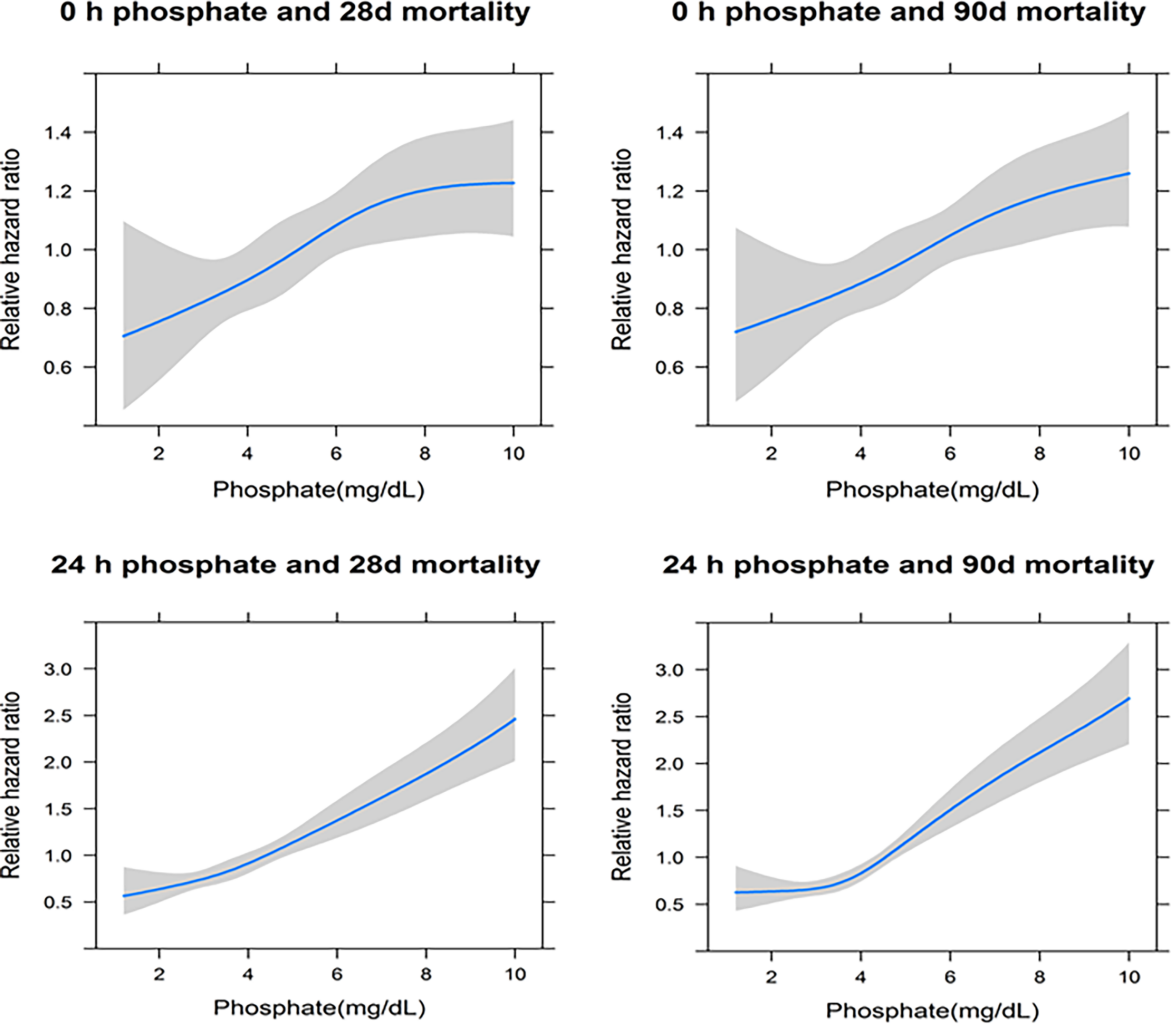
Cubic spline analysis of the associations between phosphate levels and 28- and 90-day mortality. Hazard ratios (HRs) were adjusted for age, sex, body mass index (BMI), Charlson comorbidity index (CCI), Sequential Organ Failure Assessment (SOFA) score, urine output (UO), albumin, and mean arterial pressure (MAP). Line represents HR. Shaded area represents 95% CI for the HR.

Among the 1144 patients, 710 deaths (62.1%) and 821 deaths (71.8%) occurred during 28 and 90 days, respectively. To delineate the association between phosphate and mortality, we constructed stepwise multivariable Cox models. In all three models, phosphate levels were significantly associated with an increased risk of death. The fully adjusted model (model 3) revealed that the HRs for 28- and 90-day mortality were 1.05 (per 1 mg/dL increase, 95% CI, 1.02–1.08; *P* = 0.001) and 1.05 (per 1 mg/dL increase, 95% CI, 1.02–1.08; *P* = 0.001), respectively (Table 3). Cubic spline curves after the full adjustment also showed an almost linear relationship between phosphate and mortality. This association was consistently observed when phosphate level at 24 h was entered into the analyses (Fig 2).

**Table 3:** Cox proportional hazard regression analysis for 28- and 90-day mortality.

	Phosphate at 0h as a continuous variable (Per 1 mg/dL increase)			
	28-day		90-day	
Deaths (n, %)	710 (62.1%)		821 (71.8%)	
	HR (95% CI)	*P*	HR (95% CI)	*P*
Model 1	1.06 (1.03–1.09)	<0.001	1.06 (1.03–1.09)	<0.001
Model 2	1.07 (1.04–1.10)	<0.001	1.06 (1.03–1.09)	<0.001
Model 3	1.05 (1.02–1.08)	0.001	1.05 (1.02–1.08)	0.001

Model 1: unadjusted.

Model 2: age, sex, and BMI at ICU admission.

Model 3: model 2 + CCI, SOFA score, urine output (2 h).

*Abbreviation*: *HR* Hazard ratio, *CI* Confidence interval, *BMI* Body mass index, *ICU* Intensive care unit, *CCI* Charlson comorbidity index, *SOFA* Sequential Organ Failure Assessment

### Mortality risk according to changes in phosphate levels during 24 h

**Fig 3:**
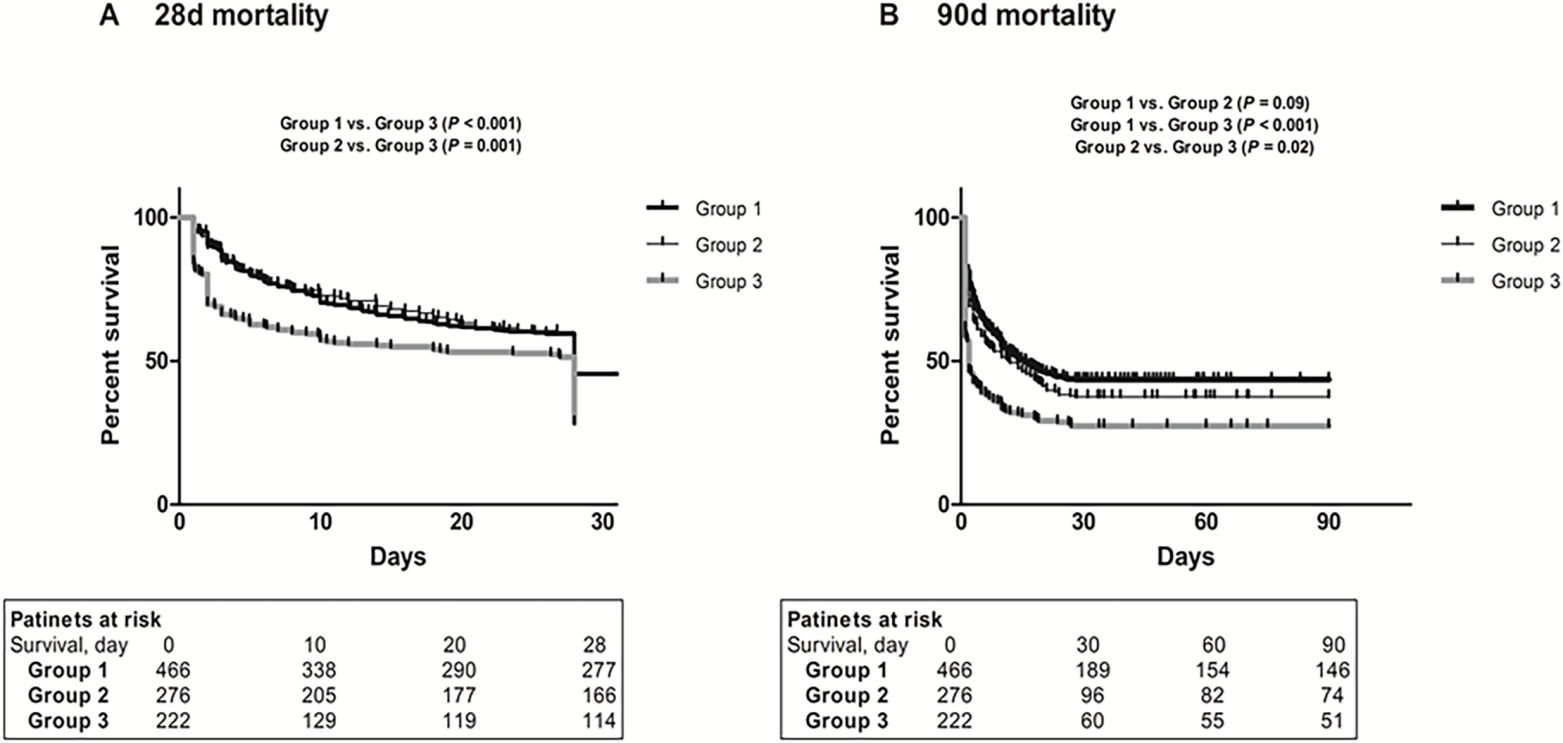
Kaplan-Meier plots for 28- and 90-day mortality according to phosphate change. Group 1 (phosphate decrease group), ≥-1.3 mg/dL decrease; group 2 (stable group), -1.3 to 0 mg/dL decrease; group 3 (phosphate increase group).

Next, we evaluated changes in phosphate levels during 24 h and further explored the significance of phosphate in these patients (S3 Table). There were 222 patients who had increased phosphate levels during 24 h. The 28- and 90-day deaths occurred more in these patients as compared with those with stable or decreased phosphate levels (28-day, 71.6% vs. 56.6%, *P* < 0.001; 90-day, 76.6% vs. 69.0%, *P* = 0.02) (Table 4). The crude HRs for 28- and 90-day mortality were 1.66 (95% CI, 1.32–2.08; *P* < 0.001) and 1.57 (95% CI, 1.27–1.94; *P* < 0.001), respectively. A Kaplan-Meier curve also confirmed that time to death was significantly shorter in patients with increased phosphate levels (Fig 3). In a Cox regression model after full adjustment, patients with increased phosphate levels conferred 1.51-fold (95% CI, 1.24–1.86; *P* < 0.001) and 1.50-fold increased risks of 28- and 90-day mortality (95% CI, 1.24–1.82; *P* < 0.001) as compared with those with decreased phosphate levels (Table 5).

**Table 4:** Death rates according to changes in phosphate levels between 0 and 24 h.

	Overall (n = 964)	Group 1 (n = 466)	Group 2 (n = 276)	Group 3(n = 222)	*P*
**28-day mortality**	579(60.1%)	254(54.5%)	166(60.1%)	159(71.6%)	<0.001
0h P < 2.5 mg/dL	13(39.4%)	0(0%)	2(25.0%)	11(44.0%)	0.30
0h P 2.5–4.5 mg/dL	159(56.2%)	26(51.0%)	65(52.4%)	68(63.0%)	0.19
0h P ≥ 4.5 mg/dL	407(62.8%)	228(54.9%)	99(68.8%)	80(89.9%)	<0.001
**90-day mortality**	682(70.7)	313(67.2%)	199(72.1%)	170(76.6%)	0.03
0h P < 2.5 mg/dL	17(51.5%)	0(0%)	4(50.0%)	13(52.0%)	0.62
0h P 2.5–4.5 mg/dL	186(65.7%)	29(56.9%)	81(65.3%)	76(70.4%)	0.24
0h P ≥ 4.5 mg/dL:	479(73.9%)	284(68.4%)	114(79.2%)	81(91.0%)	<0.001

Data are expressed as numbers (%).

Group 1 (phosphate decrease group), ≥ -1.3 mg/dL decrease; group 2 (stable group), -1.3 to 0 mg/dL decrease; group 3 (phosphate increase group)

**Table 5:** Cox proportional hazard regression analysis for 28- and 90-day mortality according to changes in phosphate levels between 0 and 24 h.

	Phosphate as a categorical variable (≥ -1.3 decrease vs. increase)^a^			
	28-day		90-day	
Deaths (n, %)	710 (62.1%)		821 (71.8%)	
	HR (95% CI)	*P*	HR (95% CI)	*P*
Model 1	1.54 (1.26–1.88)	<0.001	1.47 (1.22–1.77)	<0.001
Model 2	1.55 (1.27–1.90)	<0.001	1.47 (1.21–1.78)	<0.001
Model 3	1.51 (1.24–1.86)	<0.001	1.50 (1.24–1.82)	<0.001

a. Phosphate change “≥ -1.3 mg/dL decrease” group is a referent.

Model 1: unadjusted.

Model 2: age, sex, and BMI at ICU admission.

Model 3: model 2 + CCI, SOFA score, 2 h urine output before CRRT (mL).

*Abbreviation*: *HR* Hazard ratio, *CI* Confidence interval, *BMI* Body mass index, *ICU* Intensive care unit, *CCI* Charlson comorbidity index, *SOFA* Sequential Organ Failure Assessment.

We further examined the change of disease severity markers according to phosphate changes. The number of available data differed among the parameters. SOFA score, APACHE II score, potassium, bicarbonate, norepinephrine dose and MAP were fully evaluated except for those who expired within 24 hours. However, lactate (n = 178) and CRP level (n = 225) were not fully evaluated. The number of patients whose relevant data were fully available at 24h was 178. There was an improvement in SOFA score (*P* = 0.03), APACHE II score (*P* < 0.001), acidosis (*P* = 0.01), lactate level (*P* = 0.01), and CRP level (*P* = 0.004) in patients with decreased phosphate levels, whilst these parameters remained similar or worsened in those with increased phosphate levels (S1 Table).

### Additive prognostic value of phosphate for mortality prediction

**Fig 4:**
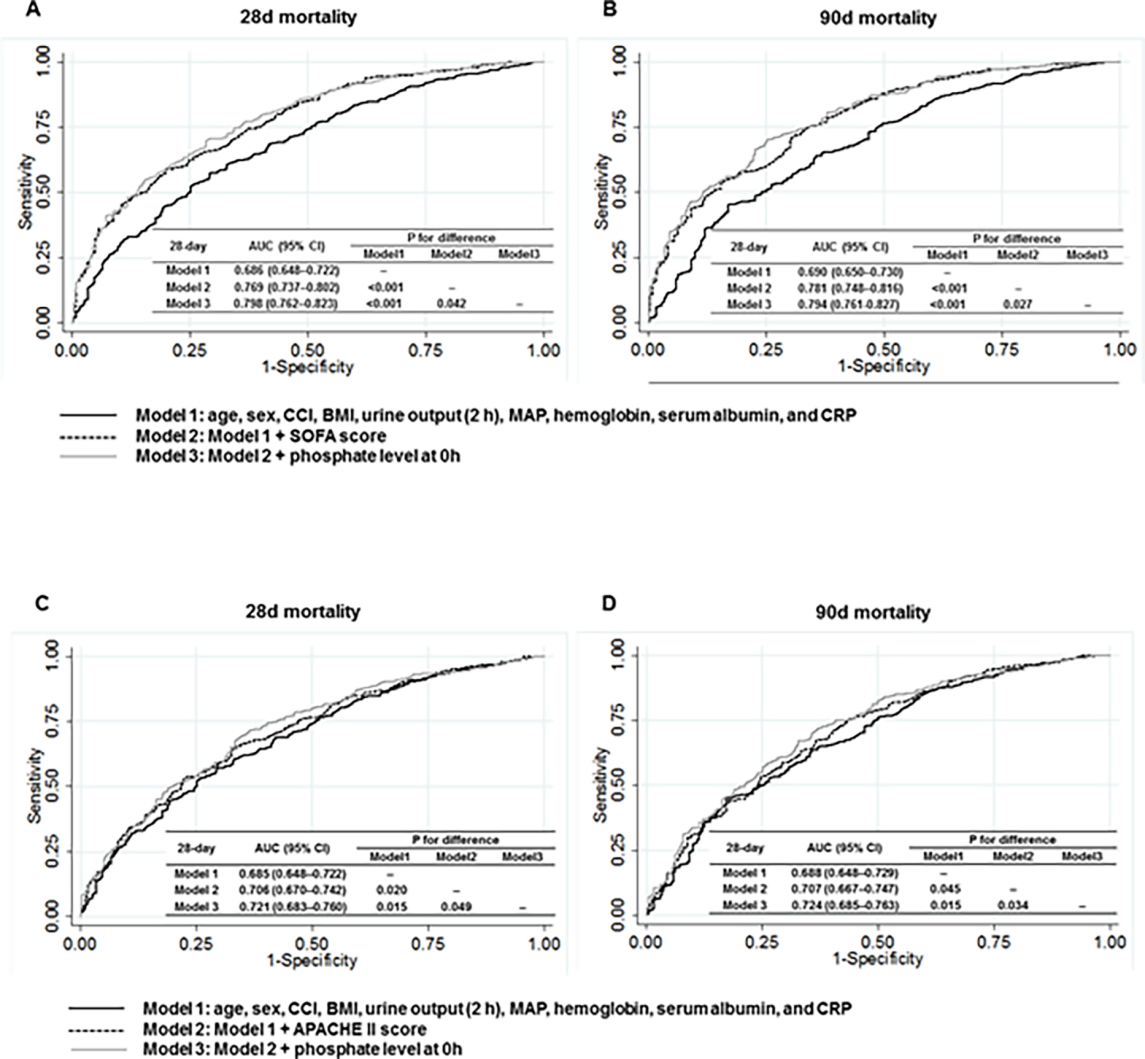
Receiver-operating characteristic plots representing the area under the curve (AUC) for the prediction of 28- and 90-daay mortality according to 0 h phosphate. The AUCs for 28- (A) and 90- day (B) mortality using models with SOFA score; The AUCs for 28- (C) and 90- day (D) mortality using models with APACHE II score. Comparison *P* values were calculated.

**Fig 5:**
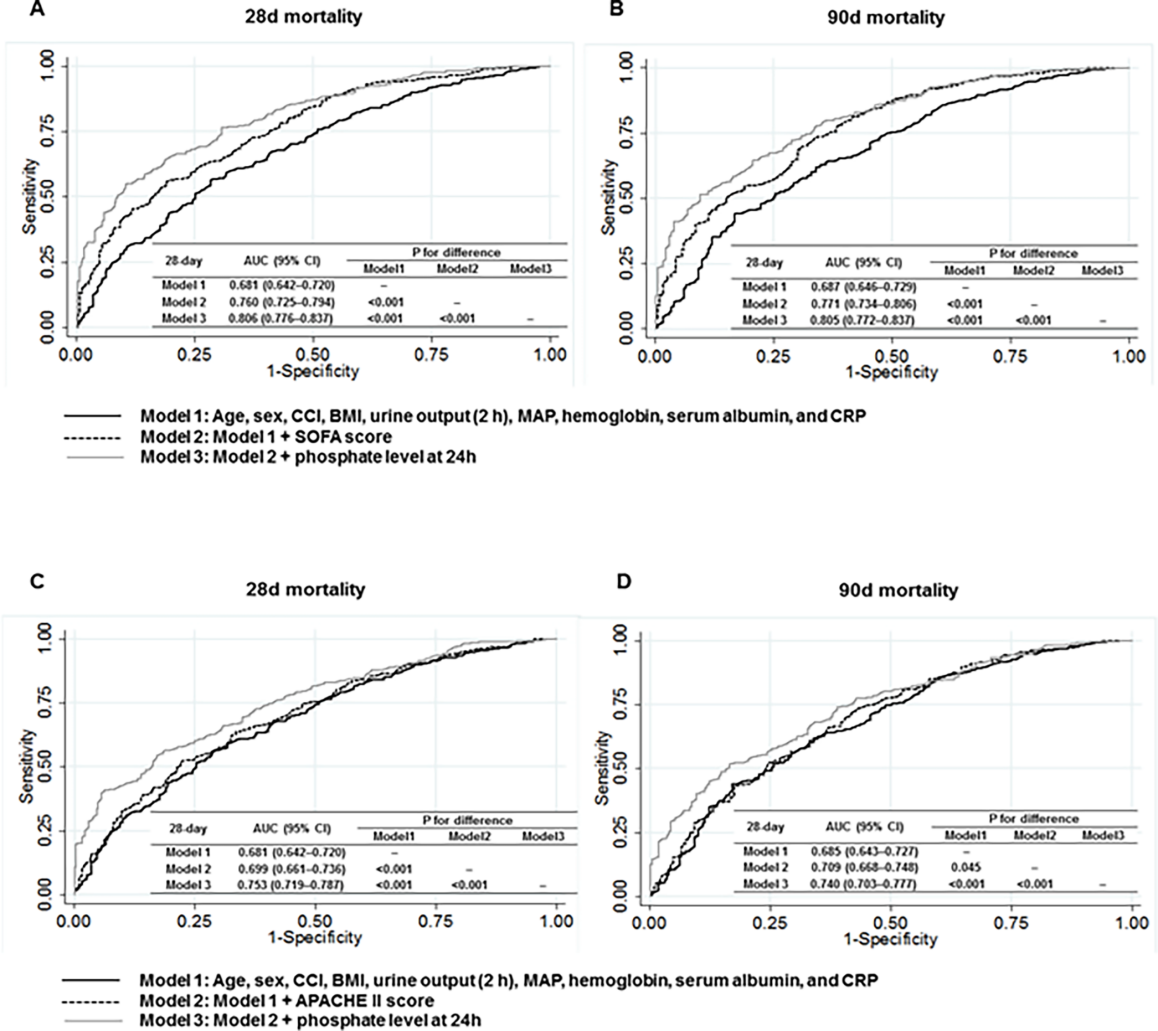
Receiver-operating characteristic plots representing the area under the curve (AUC) for the prediction of 28- and 90-day mortality according to 24 h phosphate. The AUCs for 28- (A) and 90- day (B) mortality using models with SOFA score; The AUCs for 28- (C) and 90- day (D) mortality using models with APACHE II score. Comparison *P* values were calculated.

To confirm the validity of phosphate as a useful biomarker, we calculated Harrell’s C-index for each multivariate Cox regression model. The *c*-statistics of model 1 for the prediction of 28-day mortality was 0.686 (95% CI, 0.648–0.722). In model 2 after additional adjustment for SOFA score, the *c*-statistics were significantly increased as compared with model 1 (0.769; 95% CI, 0.737–0.802; *P* <0.001). Adding phosphate to model 2 also further improved the predictive ability (0.798; 95% CI, 0.762–0.823; *P* < 0.001 vs. model 1 and *P* = 0.042 vs. model 2). This association was also found for 90-day mortality. In addition, the results remained unaltered when SOFA score was replaced with APACHE II score. These findings were further confirmed by ROC curve analyses. The area under the curve (AUC) was the largest in a model that included phosphate in addition to conventional variables and SOFA score (Fig 4). The improved predictive ability of phosphate was more evident when phosphate level at 24 h was entered into the analyses (Fig 5).

### Residual hyperphosphatemia has prognostic value in predicting mortality

As described earlier, 222 (40.6%) patients still had hyperphosphatemia at 24 h after CRRT initiation. We evaluated whether residual hyperphosphatemia can predict mortality. Similar to the analysis with phosphate level at 0 h, phosphate level at 24 h was significantly associated with a 1.15-fold increased risk of 28-day (HR, 1.15; 95% CI, 1.09–1.20; *P* < 0.001) and 90-day (HR, 1.15; 95% CI, 1.09–1.20; *P* < 0.001) mortality (S2 Table). In addition, the *c*-statistics of a model with serum phosphate significantly improved as compared with a model with conventional variables. The improved predictive ability of phosphate was more evident on ROC curve analysis when the phosphate level at 24 h was entered (Fig 4).

## Discussion

In this study, we investigated whether phosphate can serve as a biomarker of disease severity and a predictor of mortality in critically ill patients receiving CRRT. We found that phosphate level well correlates with APACHE II and SOFA scores, which are two representative scoring systems that can reflect a life-threatening condition in patients in the ICU. In addition, we demonstrated that serum phosphate is significantly associated with mortality. The prognostic value of phosphate was further verified by different statistical methods such as conventional Cox regression models, *c*-statistics analysis, and ROC curve analysis. Our robust findings suggest that phosphate is indeed a good biomarker of disease severity and is useful in risk stratification for prediction of adverse outcomes in critically ill patients.

Disturbances in electrolyte and mineral homeostasis commonly occur in patients in the ICU. These are also frequently found even in patients receiving RRT.[10] However, whether abnormalities of electrolytes and minerals are associated with disease severity is not known. We clearly showed that phosphate level significantly correlates with various clinical parameters related to disease severity. Interestingly, besides SOFA and APACHE II scores, high serum phosphate also highly associates with low blood pressure, decreased kidney function, low UO, and high acidosis level. All these parameters represent the degree of illness in the critically ill condition. In contrast, this association was not observed or weak for other electrolytes and minerals such as sodium, potassium, and calcium. Although phosphate is well cleared by dialysis therapy, we found that residual hyperphosphatemia remained in a substantial portion of patients at 24 h after CRRT. We further demonstrated that this residual hyperphosphatemia also associates with the SOFA and APACHE II scores when the phosphate level at 24 h was used in the analysis. Taken together, these findings suggest that phosphate deserves attention and can be used as a potential marker of disease severity.

We also validated the role of phosphate as a biomarker in predicting future adverse outcomes. Our previous work showed that hyperphosphatemia is significantly associated with an increased risk of death.[10] In this study, we substantiated this finding in a larger cohort by using diverse analytical methods. It should be noted that adding phosphate to conventional models significantly improved the predictive ability as compared with the models with SOFA and APACHE II scores, although the extent of the improved *c*-statistics and AUC was small. However, it is well known that the SOFA or APACHE II score encompasses almost all parameters that reflect disease severity in the critically ill condition. In fact, these two scoring systems involve a variety of organ compromises, including the cardiovascular, respiratory, hepatic, renal, and central nervous systems. Therefore, finding a novel biomarker beyond the two systems is highly challenging. Many studies have identified biomarkers in the circulation or urine to date; however, no biomarker is superior to these two scoring systems in predicting clinical adverse outcomes. For example, recent studies have suggested that urinary biomarkers such as kidney injury molecule-1 (KIM-1), *N*-acetyl-(d)-glucosaminidase (NAG), and neutrophil gelatinase-associated lipocalin (NGAL) are helpful in diagnosing AKI and in predicting mortality in various clinical settings [13–15]. Nevertheless, none of these predicts better adverse outcomes than the APACHE II score.

Cost-effectiveness is another merit of using phosphate as a biomarker. In Korea, the cost for the measurement of NGAL is 25 times higher than that for serum phosphate. Given the considerable medical cost in the management of critically ill patients in the ICU, incorporation of phosphate into risk stratification can be a cost-saving strategy. Taken all findings into account, phosphate should be highlighted as a potential biomarker of disease severity and clinical adverse outcomes.

In this study, we showed that residual hyperphosphatemia still has prognostic value. Notably, patients with a reduction in phosphate levels during 24 h had a better survival than those with stable or increased phosphate levels. This finding raises an important question on whether phosphate level should be lowered by dialysis or pharmacologic treatment in critically ill patients. Phosphate toxicity has been well recognized particularly in patients with CKD. Many epidemiologic studies have consistently shown that increased phosphate level is significantly associated with cardiovascular events and mortality in these patients [16–18]. However, there is much controversy about whether pharmacologic treatment is beneficial in improving mortality [19, 20]. The results of these studies should be interpreted with caution because the achieved levels of phosphate were not analyzed. In acute injury settings, phosphate can simply be a marker or mediator of direct toxicity. Upon cell injury, many harmful signals such as inflammatory cytokines are also released. Therefore, phosphate may not be the major culprit in the development of fatal events. Of note, increased phosphate level can elevate the fibroblast growth factor-23 (FGF23) level [21, 22]. FGF23 has emerged as a powerful biomarker of cardiovascular disease and death in patients with CKD [21, 23–25]. It is also reported to have direct toxic effects on the cardiovascular system [25–27] and to be associated with adverse outcomes in human studies with a small sample size [21, 28, 29]. By contrast, it is possible that phosphate released from dead cells is directly toxic to adjacent cells and tissues. In fact, Papa and Skulachev demonstrated that phosphate influx into cells causes mitochondrial damage and produces reactive oxygen *in vivo* [30]. Given the complex relationship between phosphate and cell death, further studies are required to elucidate the role of phosphate and the effect of phosphate-lowering treatment in critically ill patients. In addition, another possible mediator of mortality among the patients with hyperphosphatemia in the study is reduction of ionized blood calcium levels with the associated risks of neurological and cardiovascular complications such as arrhythmias.

Several limitations must be considered in this study. First, because this is a retrospective observational study, potential bias can hamper interpretation of our findings. In particular, hyperphosphatemia can occur in patients with late application of CRRT. Such different time-points of initiating CRRT can result in length-time bias. In this study, most patients started to receive CRRT within 72 hours after ICU admission. Therefore, hyperphosphatemia is unlikely caused by late treatment. Nevertheless, our observational study cannot entirely exclude length-time bias. Second, although the prescribed effluent dose was targeted to deliver ≥35 mL∙kg^-1^∙h^-1^, it is unknown whether all patients reached this target. In real clinical practice, ensuring target volume as it is prescribed is almost impossible because of many technical issues such as clotting, poor blood flow, and recirculation. To minimize this, CRRT circuit was changed every 48 hours in our center regardless of clotting status in the dialyzer filter. Third, we did not have data for parathyroid hormone, FGF23, and 1,25-dihydroxyvitamin D3 levels. In addition, other conditions that could affect phosphate level before CRRT initiation such as diet or nutritional supplement were not well captured. Because these parameters closely interact with phosphate, it would be helpful to understand the complex relationship between phosphate and its associated factors in the development of adverse outcomes if these were integrated into the analysis. Lastly, the database does not include data for recently developed biomarkers such as KIM-1, NAG, or NGAL; thus, comparative analysis between phosphate and these markers are not feasible. Forth, we showed that the predictability of phosphate for mortality was slightly better than that of the SOFA and APACHE II scores. However, the differences in AUCs were so marginal and whether such subtle but statistically significant difference can have clinical impact has been under debate [31–33]. As mentioned earlier, these two scoring systems are the most powerful assessment tools that can predict adverse outcomes in patients in the ICU, thus improving predictive ability using some potential markers is not easy beyond these two scoring systems. In this regard, phosphate should receive more attention given its advantages of predictability and cost-effectiveness. In conclusion, we demonstrated that phosphate is a potential biomarker that can reflect disease severity and predict mortality in critically ill patients receiving CRRT. Future studies should address whether phosphate should be incorporated into risk stratification and whether lowering the phosphate level can reduce mortality in these patients.

## Supporting information

S1 Table Changes of disease severity markers according to phosphate changes. (DOCX)

S2 Table Cox proportional hazard regression analysis for 28- and 90-day mortality in 399 patients who survived 24 h after continuous renal replacement therapy initiation. (DOCX)

S3 Table Baseline characteristics according to changes in phosphate levels between 0 and 24 h. (DOCX)

## References

[1] Uchino S, Kellum JA, Bellomo R, Doig GS, Morimatsu H, Morgera S, et al Acute renal failure in critically ill patients: a multinational, multicenter study. JAMA. 2005;294(7):813–8. doi: 10.1001/jama.294.7.813 . 16106006 10.1001/jama.294.7.813

[2] Chawla LS, Kimmel PL. Acute kidney injury and chronic kidney disease: an integrated clinical syndrome. Kidney Int. 2012;82(5):516–24. doi: 10.1038/ki.2012.208 . 22673882 10.1038/ki.2012.208

[3] Investigators RRTS, Bellomo R, Cass A, Cole L, Finfer S, Gallagher M, et al Intensity of continuous renal-replacement therapy in critically ill patients. N Engl J Med. 2009;361(17):1627–38. doi: 10.1056/NEJMoa0902413 . 19846848 10.1056/NEJMoa0902413

[4] Gaudry S, Hajage D, Schortgen F, Martin-Lefevre L, Pons B, Boulet E, et al Initiation Strategies for Renal-Replacement Therapy in the Intensive Care Unit. N Engl J Med. 2016;375(2):122–33. doi: 10.1056/NEJMoa1603017 . 27181456 10.1056/NEJMoa1603017

[5] Demirjian S, Chertow GM, Zhang JH, O'Connor TZ, Vitale J, Paganini EP, et al Model to predict mortality in critically ill adults with acute kidney injury. Clin J Am Soc Nephrol. 2011;6(9):2114–20. doi: 10.2215/CJN.02900311 ; PubMed Central PMCID: PMCPMC3359007. 21896828 10.2215/CJN.02900311 PMC3359007

[6] Buckley MS, Leblanc JM, Cawley MJ. Electrolyte disturbances associated with commonly prescribed medications in the intensive care unit. Crit Care Med. 2010;38(6 Suppl):S253–64. doi: 10.1097/CCM.0b013e3181dda0be . 20502178 10.1097/CCM.0b013e3181dda0be

[7] Peppers MP, Geheb M, Desai T. Endocrine crises. Hypophosphatemia and hyperphosphatemia. Crit Care Clin. 1991;7(1):201–14. . 2007215

[8] Foley RN. Phosphate levels and cardiovascular disease in the general population. Clin J Am Soc Nephrol. 2009;4(6):1136–9. doi: 10.2215/CJN.01660309 . 19423568 10.2215/CJN.01660309

[9] Sullivan C, Sayre SS, Leon JB, Machekano R, Love TE, Porter D, et al Effect of food additives on hyperphosphatemia among patients with end-stage renal disease: a randomized controlled trial. JAMA. 2009;301(6):629–35. doi: 10.1001/jama.2009.96 . 19211470 10.1001/jama.2009.96

[10] Jung SY, Kim H, Park S, Jhee JH, Yun HR, Kim H, et al Electrolyte and mineral disturbances in septic acute kidney injury patients undergoing continuous renal replacement therapy. Medicine (Baltimore). 2016;95(36):e4542 doi: 10.1097/MD.0000000000004542 ; PubMed Central PMCID: PMCPMC5023866. 27603344 10.1097/MD.0000000000004542 PMC5023866

[11] Ozmen S, Danis R, Akin D, Cil T, Yazanel O. Parathyroid hormone as a marker for the differential diagnosis of acute and chronic renal failure. Ren Fail. 2007;29(4):509–12. doi: 10.1080/08860220701275006 . 17497477 10.1080/08860220701275006

[12] Mehta RL, Kellum JA, Shah SV, Molitoris BA, Ronco C, Warnock DG, et al Acute Kidney Injury Network: report of an initiative to improve outcomes in acute kidney injury. Crit Care. 2007;11(2):R31 doi: 10.1186/cc5713 ; PubMed Central PMCID: PMCPMC2206446. 17331245 10.1186/cc5713 PMC2206446

[13] Liangos O, Perianayagam MC, Vaidya VS, Han WK, Wald R, Tighiouart H, et al Urinary N-acetyl-beta-(D)-glucosaminidase activity and kidney injury molecule-1 level are associated with adverse outcomes in acute renal failure. J Am Soc Nephrol. 2007;18(3):904–12. doi: 10.1681/ASN.2006030221 . 17267747 10.1681/ASN.2006030221

[14] Sanjeevani S, Pruthi S, Kalra S, Goel A, Kalra OP. Role of neutrophil gelatinase-associated lipocalin for early detection of acute kidney injury. Int J Crit Illn Inj Sci. 2014;4(3):223–8. doi: 10.4103/2229-5151.141420 ; PubMed Central PMCID: PMCPMC4200548. 25337484 10.4103/2229-5151.141420 PMC4200548

[15] Haase M, Bellomo R, Devarajan P, Schlattmann P, Haase-Fielitz A, Group NM-aI. Accuracy of neutrophil gelatinase-associated lipocalin (NGAL) in diagnosis and prognosis in acute kidney injury: a systematic review and meta-analysis. Am J Kidney Dis. 2009;54(6):1012–24. doi: 10.1053/j.ajkd.2009.07.020 . 19850388 10.1053/j.ajkd.2009.07.020

[16] Block GA, Hulbert-Shearon TE, Levin NW, Port FK. Association of serum phosphorus and calcium x phosphate product with mortality risk in chronic hemodialysis patients: a national study. Am J Kidney Dis. 1998;31(4):607–17. . 9531176 10.1053/ajkd.1998.v31.pm9531176

[17] Ganesh SK, Stack AG, Levin NW, Hulbert-Shearon T, Port FK. Association of elevated serum PO(4), Ca x PO(4) product, and parathyroid hormone with cardiac mortality risk in chronic hemodialysis patients. J Am Soc Nephrol. 2001;12(10):2131–8. . 11562412 10.1681/ASN.V12102131

[18] Kestenbaum B, Sampson JN, Rudser KD, Patterson DJ, Seliger SL, Young B, et al Serum phosphate levels and mortality risk among people with chronic kidney disease. J Am Soc Nephrol. 2005;16(2):520–8. doi: 10.1681/ASN.2004070602 . 15615819 10.1681/ASN.2004070602

[19] Palmer SC, Gardner S, Tonelli M, Mavridis D, Johnson DW, Craig JC, et al Phosphate-Binding Agents in Adults With CKD: A Network Meta-analysis of Randomized Trials. Am J Kidney Dis. 2016;68(5):691–702. doi: 10.1053/j.ajkd.2016.05.015 . 27461851 10.1053/j.ajkd.2016.05.015

[20] Isakova T, Gutierrez OM, Chang Y, Shah A, Tamez H, Smith K, et al Phosphorus binders and survival on hemodialysis. J Am Soc Nephrol. 2009;20(2):388–96. doi: 10.1681/ASN.2008060609 ; PubMed Central PMCID: PMCPMC2637053. 19092121 10.1681/ASN.2008060609 PMC2637053

[21] Leaf DE, Wolf M, Waikar SS, Chase H, Christov M, Cremers S, et al FGF-23 levels in patients with AKI and risk of adverse outcomes. Clin J Am Soc Nephrol. 2012;7(8):1217–23. doi: 10.2215/CJN.00550112 ; PubMed Central PMCID: PMCPMC3408118. 22700885 10.2215/CJN.00550112 PMC3408118

[22] Titan SM, Zatz R, Graciolli FG, dos Reis LM, Barros RT, Jorgetti V, et al FGF-23 as a predictor of renal outcome in diabetic nephropathy. Clin J Am Soc Nephrol. 2011;6(2):241–7. doi: 10.2215/CJN.04250510 ; PubMed Central PMCID: PMCPMC3052212. 20966122 10.2215/CJN.04250510 PMC3052212

[23] Leaf DE, Jacob KA, Srivastava A, Chen ME, Christov M, Juppner H, et al Fibroblast Growth Factor 23 Levels Associate with AKI and Death in Critical Illness. J Am Soc Nephrol. 2016 doi: 10.1681/ASN.2016080836 . 28028134 10.1681/ASN.2016080836 PMC5461795

[24] Gutierrez OM, Mannstadt M, Isakova T, Rauh-Hain JA, Tamez H, Shah A, et al Fibroblast growth factor 23 and mortality among patients undergoing hemodialysis. N Engl J Med. 2008;359(6):584–92. doi: 10.1056/NEJMoa0706130 ; PubMed Central PMCID: PMCPMC2890264. 18687639 10.1056/NEJMoa0706130 PMC2890264

[25] Isakova T, Xie H, Yang W, Xie D, Anderson AH, Scialla J, et al Fibroblast growth factor 23 and risks of mortality and end-stage renal disease in patients with chronic kidney disease. JAMA. 2011;305(23):2432–9. doi: 10.1001/jama.2011.826 ; PubMed Central PMCID: PMCPMC3124770. 21673295 10.1001/jama.2011.826 PMC3124770

[26] Faul C, Amaral AP, Oskouei B, Hu MC, Sloan A, Isakova T, et al FGF23 induces left ventricular hypertrophy. J Clin Invest. 2011;121(11):4393–408. doi: 10.1172/JCI46122 ; PubMed Central PMCID: PMCPMC3204831. 21985788 10.1172/JCI46122 PMC3204831

[27] Jimbo R, Kawakami-Mori F, Mu S, Hirohama D, Majtan B, Shimizu Y, et al Fibroblast growth factor 23 accelerates phosphate-induced vascular calcification in the absence of Klotho deficiency. Kidney Int. 2014;85(5):1103–11. doi: 10.1038/ki.2013.332 . 24088960 10.1038/ki.2013.332

[28] Zhang M, Hsu R, Hsu CY, Kordesch K, Nicasio E, Cortez A, et al FGF-23 and PTH levels in patients with acute kidney injury: A cross-sectional case series study. Ann Intensive Care. 2011;1(1):21 doi: 10.1186/2110-5820-1-21 ; PubMed Central PMCID: PMCPMC3224491. 21906363 10.1186/2110-5820-1-21 PMC3224491

[29] Ali FN, Hassinger A, Price H, Langman CB. Preoperative plasma FGF23 levels predict acute kidney injury in children: results of a pilot study. Pediatr Nephrol. 2013;28(6):959–62. doi: 10.1007/s00467-012-2395-2 . 23314442 10.1007/s00467-012-2395-2

[30] Papa S, Skulachev VP. Reactive oxygen species, mitochondria, apoptosis and aging. Mol Cell Biochem. 1997;174(1–2):305–19. . 9309704

[31] Baker SG, Schuit E, Steyerberg EW, Pencina MJ, Vickers A, Moons KG, et al How to interpret a small increase in AUC with an additional risk prediction marker: decision analysis comes through. Stat Med. 2014;33(22):3946–59. doi: 10.1002/sim.6195 ; PubMed Central PMCID: PMCPMC4156533. 24825728 10.1002/sim.6195 PMC4156533

[32] Cook NR. Use and misuse of the receiver operating characteristic curve in risk prediction. Circulation. 2007;115(7):928–35. doi: 10.1161/CIRCULATIONAHA.106.672402 . 17309939 10.1161/CIRCULATIONAHA.106.672402

[33] Gail MH. Discriminatory accuracy from single-nucleotide polymorphisms in models to predict breast cancer risk. J Natl Cancer Inst. 2008;100(14):1037–41. doi: 10.1093/jnci/djn180 ; PubMed Central PMCID: PMCPMC2528005. 18612136 10.1093/jnci/djn180 PMC2528005

