# Supplementary material for: Phosphate is a potential biomarker of disease severity and predicts adverse outcomes in acute kidney injury patients undergoing continuous renal replacement therapy
Source: PLoS One. 2018 Feb 7;13(2):e0191290. doi: 10.1371/journal.pone.0191290 (PMC5802883; doi:10.1371/journal.pone.0191290)
Supplement: S1 Table — (DOCX) [file pone.0191290.s001.docx]

**S1 TABLE.** Changes of disease severity markers according to phosphate changes

| Variables | Group 1 | Group 2 | Group 3 | *P* |
| --- | --- | --- | --- | --- |
| Delta urine output (n=852) | -10.0 [-50.0–0] | -10.5 [-75.0–0] | -15.0 [-30.0–0] | 0.86 |
| Delta SOFA score (n=964) | -1.0 [-3.0–0] | -1.0 [-2.0–0] | 0 [-2.0–2.0] | 0.03 |
| Delta APACHE II score (n=964) | -6.0 [-10.0–-2.0] | -3.0 [-7.0–0] | -1.0 [-4.0–2.0] | 0.001 |
| Delta norepinephrine dose (n=783) | -0.02 [-0.1–0.03] | -0.003 [-0.1–0.003] | 0 [-0.4–0.1] | 0.89 |
| Delta MAP (mmHg) (n=964) | 2.0 [-10.0–12.3] | 8.0 [-7.0–17.6] | 8.6 [-6.3–15.3] | 0.53 |
| Delta potassium (mmol/L) (n=964) | -0.8 [-1.6–0.1] | -0.2 [-0.8–0.6] | -0.6 [-1.1–0.3] | 0.02 |
| Delta bicarbonate (mmol/L) (n=964) | 3.0 [0–6.0] | 1.0 [-2.0–5.0] | 0 [-3.0–2.5] | 0.01 |
| Delta lactate (mg/dL) (n=178) | -0.3 [-3.3–0.7] | 0.5 [-0.8–2.7] | 0.7 [-0.2–3.0] | 0.01 |
| Delta CRP (mg/L) (n=225) | -0.8 [-3.0–2.6] | 2.7 [-4.5–7.9] | 5.8 [0.8–19.8] | 0.004 |

Data are expressed as median (interquartile range)

Group 1 (phosphate decrease group), ≥ -1.3 mg/dL decrease; group 2 (stable group), -1.3 to 0 mg/dL decrease; group 3 (phosphate increase group)

Delta = 24 h- 0 h data

*Abbreviation:* *SOFA* Sequential Organ Failure Assessment, *APACHE II* Acute Physiology and Chronic Health Evaluation II, *MAP* Mean arterial pressure, *CRP*, C-reactive protein
