# Supplementary material for: Phosphate is a potential biomarker of disease severity and predicts adverse outcomes in acute kidney injury patients undergoing continuous renal replacement therapy
Source: PLoS One. 2018 Feb 7;13(2):e0191290. doi: 10.1371/journal.pone.0191290 (PMC5802883; doi:10.1371/journal.pone.0191290)
Supplement: S2 Table — (DOCX) [file pone.0191290.s002.docx]

**S2 TABLE.** Cox proportional hazard regression analysis for 28- and 90-day mortality in 399 patients who survived 24 h after continuous renal replacement therapy initiation

|  | Phosphate as a continuous variable  (Per 1 mg/dL increase) | | | |
| --- | --- | --- | --- | --- |
| Model | **28-day** | | **90-day** | |
|  | HR (95% CI) | *P* | HR (95% CI) | *P* |
| Model 1 | 1.12 (1.08–1.17) | <0.001 | 1.13 (1.08–1.17) | <0.001 |
| Model 2 | 1.17 (1.12–1.23) | <0.001 | 1.17 (1.12–1.23) | <0.001 |
| Model 3 | 1.15 (1.09–1.20) | <0.001 | 1.15 (1.09–1.20) | <0.001 |

Model 1: unadjusted.

Model 2: age, sex, and BMI at ICU admission.

Model 3: model 2 + CCI, SOFA score, and urine output (2 h)

*Abbreviations:* *HR* hazard ratio, *CI* confidence interval, *BMI* body mass index, ICU intensive care unit, *CCI* Charlson comorbidity index, *SOFA* Sequential Organ Failure Assessment
