# Supplementary material for: Phosphate is a potential biomarker of disease severity and predicts adverse outcomes in acute kidney injury patients undergoing continuous renal replacement therapy
Source: PLoS One. 2018 Feb 7;13(2):e0191290. doi: 10.1371/journal.pone.0191290 (PMC5802883; doi:10.1371/journal.pone.0191290)
Supplement: S3 Table — (DOCX) [file pone.0191290.s003.docx]

**S3 TABLE.** Baseline characteristics according to changes in phosphate levels between 0 and 24 h

| Patient Characteristics | Group 1  (n=466) | Group 2  (n=276) | Group 3  (n=222) | | *P* |  |
| --- | --- | --- | --- | --- | --- | --- |
| Age (years) | 63.2 ± 14.5 | 64.3 ± 13.5 | 61.6 ± 15.3 | | 0.12 |  |
| Male (%) | 281 (60.3) | 171 (62.0) | 140 (63.1) | | 0.77 |  |
| Hypertension (%) | 253 (54.3) | 152 (55.1) | 119 (53.6) | | 0.95 |  |
| Diabetes mellitus (%) | 168 (36.1) | 106 (38.4) | 73 (32.9) | | 0.44 |  |
| Heart failure (%) | 82 (17.6) | 50 (18.1) | 34 (15.3) | | 0.68 |  |
| Myocardial infarction (%) | 37 (7.9) | 32 (11.6) | 18 (8.1) | | 0.21 |  |
| Cerebrovascular disease (%) | 49 (10.6) | 25 (9.1) | 30 (13.6) | | 0.27 |  |
| COPD (%) | 34 (7.3) | 21 (7.6) | 15 (6.8) | | 0.94 |  |
| MV (%) | 357 (76.6) | 203 (73.6) | 179 (81.0) | | 0.15 |  |
| CCI | 3.1 ± 2.2 | 3.3 ± 2.4 | 2.9 ± 2.2 | | 0.10 |  |
| Cause of AKI |  |  |  | | 0.58 |  |
| Sepsis | 330 (70.8) | 208 (75.4) | 172 (77.5) | | 0.13 |  |
| Nephrotoxin | 13 (2.8) | 11 (4.0) | 7 (3.2) | | 0.67 |  |
| Ischemia | 41 (8.8) | 18 (6.5) | 15 (6.8) | | 0.45 |  |
| Surgery | 37 (7.9) | 20 (7.2) | 13 (5.9) | | 0.62 |  |
| Others | 45 (9.7) | 19 (6.9) | 15 (6.8) | | 0.28 |  |
| Cause of CRRT |  |  |  | | 0.66 |  |
| Volume overload (%) | 56 (12.0) | 27 (9.8) | 27 (12.2) | | 0.60 |  |
| Metabolic acidosis (%) | 95 (20.4) | 56 (20.3) | 50 (22.5) | | 0.78 |  |
| Hyperkalemia (%) | 24 (5.2) | 13 (4.7) | 7 (3.2) | | 0.50 |  |
| Uremia (%) | 54 (11.6) | 21 (7.6) | 22 (9.9) | | 0.22 |  |
| Oliguria (%) | 120 (25.8) | 86 (31.2) | 57 (25.7) | | 0.23 |  |
| Others (%) | 117 (25.1) | 73 (26.4) | 59 (26.6) | | 0.88 |  |
| Duration from diagnosis of AKI to CRRT (h) | 0.9 [0.2–5.7] | 1.2 [0.2–6.0] | 0.6 [0.1–3.4] | | 0.58 |  |
| AKIN stages |  |  |  |  | 0.01 |  |
| Stage 2 (%) | 103 (22.1) | 79 (28.6) | 73 (32.9) | |  |  |
| Stage 3 (%) | 363 (77.9) | 197 (71.4) | 149 (67.1) | |  |  |
| BMI (kg/m^2^)  at ICU admission | 24.0 ± 4.5 | 23.7 ± 4.8 | 24.1 ± 4.5 | | 0.68 |  |
| SOFA score | 11.9 ± 3.6 | 11.6 ± 3.5 | 12.3 ± 3.1 | | 0.08 |  |
| APACHE II score | 27.6 ± 8.1 | 26.3 ± 8.1 | 27.2 ± 8.0 | | 0.08 |  |
| SBP (mmHg) | 114.3 ± 20.4 | 112.2 ± 20.7 | 109.9 ± 22.2 | | 0.04 |  |
| DBP (mmHg) | 62.1 ± 14.7 | 59.8 ± 13.3 | 60.8 ± 13.5 | | 0.09 |  |
| MAP (mmHg) | 83.6 ± 15.5 | 85.8 ± 17.5 | 79.9 ± 13.8 | | 0.25 |  |
| Hemoglobin (g/dL) | 9.7 ± 2.3 | 9.6 ± 2.1 | 9.7 ± 2.3 | | 0.92 |  |
| White blood cell (μL) | 12960 [7940–19710] | 10720 [5632–17012] | 10620 [4740–18765] | | 0.16 |  |
| Albumin (g/dL) | 2.6 ± 0.6 | 2.6 ± 0.6 | 2.5 ± 0.6 | | 0.004 |  |
| Potassium (mEq/L) | 4.8 ± 1.1 | 4.6 ± 1.1 | 4.7 ± 1.1 | | 0.08 |  |
| Bicarbonate (mEq/L) | 16.1 ± 5.4 | 17.7 ± 5.4 | 16.7 ± 6.1 | | 0.002 |  |
| BUN (mg/dL) | 58.4 ± 32.2 | 56.8 ± 29.8 | 50.7 ± 25.3 | | 0.007 |  |
| Phosphate (mg/dL) | 6.8 ± 2.3 | 4.9 ± 2.0 | 4.6 ± 2.2 | | <0.001 |  |
| Creatinine (mg/dL) | 3.0 ± 1.9 | 2.6 ± 1.4 | 2.3 ± 1.2 | | <0.001 |  |
| CRRT dose (ml/kg) | 36.6 ± 4.3 | 36.3 ± 4.3 | 36.8 ± 5.1 | | 0.42 |  |
| CRP (mg/L) | 63.6 [17.9–153.0] | 79.8 [21.0–180.7] | 96.0 [27.1–201.1] | | 0.02 |  |

Data are expressed as mean ± standard deviations, median (interquartile range), or number (%).

All laboratory measurements were done at 0 h (before starting CRRT)

Group 1 (phosphate decrease group), ≥ -1.3 mg/dL decrease; group 2 (stable group), -1.3 to 0 mg/dL decrease; group 3 (phosphate increase group)

*Abbreviations:* *COPD* Chronic obstructive pulmonary disease, *CCI* Charlson comorbidity index, *BMI* Body mass index, *AKIN* Acute kidney injury criteria, *ICU* Intensive care unit, *CRRT* Continuous renal replacement therapy, *eGFR* estimated glomerular filtration rate, *SOFA* Sequential Organ Failure Assessment Score, *APACHE II* Acute Physiology and Chronic Health Evaluation II, *SBP* Systolic blood pressure, *DBP* Diastolic blood pressure, *MAP* Mean arterial pressure, *CRP* C-reactive protein
